# Targeted Vaginal Microbiome Modulation by Postbiotic Intervention Reduces Dysbiosis-Associated Pathogens in Infertile Women: A Pilot Study

**DOI:** 10.7150/ijms.130288

**Published:** 2026-04-16

**Authors:** Li-Te Lin, Chia-Jung Li, Pei-Hsuan Lin, Shih-Hsuan Cheng, Emmanuel Naveen Raj, Yu-Chen Chen, Chyi-Uei Chern, Zhi-Hong Wen, Kuan-Hao Tsui

**Affiliations:** 1 Department of Obstetrics and Gynaecology, Kaohsiung Veterans General Hospital, Kaohsiung 813, Taiwan.; 2 Center of General Education, Cheng Shiu University, Kaohsiung 833, Taiwan.; 3 Department of Biological Science, National Sun Yat-sen University, Kaohsiung 804, Taiwan.; 4 Institute of Biopharmaceutical Sciences, National Sun Yat-sen University, Kaohsiung 804, Taiwan.; 5 School of Medicine, College of Medicine, National Sun Yat-sen University, Kaohsiung 804, Taiwan.; 6 Department of Obstetrics and Gynaecology, National Yang-Ming University School of Medicine, Taipei 112, Taiwan.; 7 Department of Marine Biotechnology and Resources, National Sun Yat-sen University, Kaohsiung 804, Taiwan.; 8 National Museum of Marine Biology & Aquarium, Pingtung 944, Taiwan.; 9 Department of Obstetrics and Gynecology, Taipei Veterans General Hospital, Taipei 112, Taiwan.; 10 Department of Medicine, Tri-Service General Hospital, National Defense Medical Center, Taipei 114, Taiwan.

**Keywords:** vaginal microbiome, postbiotics, antimicrobial peptides, infertility, assisted reproductive technology, *in vitro* fertilization

## Abstract

Background: Vaginal dysbiosis characterized by depleted Lactobacillus populations is associated with impaired fertility outcomes in women undergoing assisted reproduction. Postbiotic interventions delivering antimicrobial peptides without live bacteria may offer targeted microecological modulation while preserving community stability. We investigated the microbial and clinical effects of an eight-week antimicrobial peptide-based postbiotic in infertile women.

Methods: Fifteen infertile women (mean age 38.7 ± 4.1 years) with ≥ 2 prior *in vitro* fertilization failures received intravaginal postbiotic therapy (VAGINNE^®^, Good-Care Biotech, Ltd.) for 8.0 ± 0.3 weeks before frozen embryo transfer (FET). Paired vaginal samples collected pre- and post-treatment underwent full-length 16S rRNA gene sequencing using PacBio Sequel platform. Microbiome composition, beta-diversity, differential abundance, and absolute bacterial load were analyzed.

Results: The cohort achieved a clinical pregnancy rate of 46.7% (7/15) following postbiotic intervention and subsequent FET. Beta-diversity analysis revealed preservation of global community structure (PERMANOVA R² = 0.05, p = 0.65), indicating ecological stability. Despite cohort-level stability, patient-specific analysis identified favorable microbiome restructuring in 73% of participants (11/15), including emergence of Lactobacillus-dominant communities in previously dysbiotic profiles. Absolute abundance quantification demonstrated selective elimination of dysbiosis-associated pathogens: Prevotella decreased from 408.47±990.78 to 0.29±0.83 cells/sample (p < 0.01), Pseudomonas from 419.07±567.04 to 89.14±114.42 (p < 0.05), and complete clearance of Howardella (3.53±9.87 to undetectable). Conversely, Gluconacetobacter increased significantly from undetectable to 2.93±4.18 cells/sample (p < 0.05).

Conclusions: In this pilot cohort, antimicrobial peptide-based postbiotic therapy was associated with encouraging pregnancy outcomes and targeted pathogen clearance. The intervention selectively reduced high-burden detrimental taxa while maintaining microbiome architectural integrity, supporting precision microecological modulation as a promising adjuvant strategy for reproductive health.

## Introduction

Infertility affects approximately 15% of reproductive-aged couples worldwide and remains a major clinical challenge, particularly among women of advanced maternal age in whom both ovarian and reproductive tract factors contribute to reduced fecundity 1, 2. Although traditional etiologies such as ovulatory dysfunction, tubal disease, endometriosis, and diminished ovarian reserve account for many cases, a substantial proportion of women experience unexplained infertility or recurrent implantation failure despite comprehensive evaluation 3, 4. Emerging evidence suggests that the female reproductive tract microbiome, particularly vaginal and endometrial microbial communities, plays an important and previously underrecognized role in reproductive competence 5-7.

The healthy vaginal ecosystem is typically dominated by Lactobacillus species (L. crispatus, L. gasseri, L. jensenii, L. iners) that maintain protective acidity through lactic acid production and synthesis of antimicrobial compounds 8 . This Lactobacillus-dominant state, corresponding to Community State Types (CST) I, II, III, and V, correlates with favorable fertility outcomes 9. In contrast, CST-IV characterized by Lactobacillus depletion and polymicrobial communities enriched in Gardnerella, Prevotella, Atopobium, and anaerobes represents vaginal dysbiosis associated with inflammation, bacterial vaginosis, and compromised reproductive success 7, 10.

Multiple studies demonstrate that women with non-Lactobacillus-dominant microbiota exhibit elevated rates of implantation failure and pregnancy loss. Large-scale analyses suggest that Lactobacillus-rich vaginal microbiomes correlate with higher implantation and ongoing pregnancy rates, whereas abundance of pathogenic genera including Prevotella, Pseudomonas, Ralstonia, and Gardnerella predict implantation failure 11-15. Similar associations persist across recurrent implantation failure cohorts and aberrant endometrial microbiome profiles 12, 16, 17.

These observations motivate microbiome-targeted interventions to restore beneficial Lactobacillus dominance. Current strategies include antibiotics, probiotics, and vaginal microbiota transplantation 18. However, antibiotic approaches provide transient dysbiosis suppression while risking antimicrobial resistance and secondary imbalances 19. Probiotic efficacy remains uncertain given variable colonization success in vaginal microenvironments 20.

Postbiotics, defined as beneficial bioactive compounds derived from microbial metabolism, include antimicrobial peptides, organic acids, and cell wall components. These formulations offer theoretical advantages over live probiotics, including enhanced stability, improved safety profiles, selective antimicrobial activity without requiring live bacterial colonization, and preservation of the broader ecological structure of the host microbiome 21-23. Despite promising rationale, clinical evidence for postbiotic applications in reproductive tract dysbiosis remains limited.

Based on this rationale, we hypothesized that antimicrobial peptide-based postbiotics could achieve targeted clearance of high-burden dysbiosis-associated genera while preserving overall microbiome stability. To test this hypothesis, we conducted a pilot study using paired full-length 16S rRNA gene sequencing to investigate an eight-week postbiotic intervention in women with advanced maternal age and recurrent *in vitro* fertilization (IVF) failure, with assessment of vaginal microbiome composition, taxonomic abundance changes, and clinical pregnancy outcomes.

## Methods

### Study design and patient selection

This prospective pilot study was conducted at the Reproductive Center, Kaohsiung Veterans General Hospital, Taiwan, between April and July 2025. Fifteen infertile women were enrolled prior to planned frozen embryo transfer (FET) following the IVF protocol. Women were eligible if they met all of the following: (i) age 30-45 years; (ii) body mass index 18-30 kg/m²; (iii) history of ≥2 prior IVF treatment failures despite transfer of good-quality embryos; and (iv) willingness to comply with the eight-week postbiotic intervention protocol. Women were excluded if they had any of the following: (i) active acute vaginal infection with symptomatic presentation; (ii) antibiotic or probiotic use within the preceding three months; (iii) congenital uterine anomalies including septate, bicornuate, or unicornuate uterus; (iv) severe intrauterine adhesions (Asherman syndrome grade III-IV); (v) history of gynecologic malignancy; (vi) known allergy or hypersensitivity to Lactobacillus-derived products; or (vii) current ongoing pregnancy.

All participants received intravaginal postbiotic therapy (VAGINNE^®^, Good-Care Biotech, Ltd.) for eight consecutive weeks. Vaginal swab specimens were obtained at two standardized time points: baseline (before initiating intervention) and post-treatment (after completing eight-week intervention). Written informed consent was obtained from all participants. Demographic characteristics, menstrual history, infertility parameters, and clinical outcomes were extracted from electronic medical records. As a pilot feasibility study, a pragmatic sample size of fifteen participants was selected to provide preliminary effect estimates for future powered trials.

### Ethics statement

This study was approved by the Institutional Review Board of Kaohsiung Veterans General Hospital (approval number: KSVGH25-CT1-18) and conducted in accordance with the Declaration of Helsinki and Good Clinical Practice guidelines. All participants provided written informed consent prior to enrollment. The trial was registered with ClinicalTrials.gov under the identifier NCT07278024. During manuscript preparation, the authors used an AI language model (Claude Sonnet 4.5, Anthropic) to enhance readability and language clarity. The authors subsequently reviewed and edited all content and take full responsibility for the publication.

### Postbiotic preparation and composition

VAGINNE^®^ (Good-Care Biotech, Ltd.) is a cell-free fermentation supernatant derived from three Lactobacillus species: L. crispatus, L. gasseri, and L. jensenii. The manufacturing process involves controlled fermentation in bovine milk medium at 30-40 °C for 16-18 hours with chitin supplementation to enhance Lactobacillus growth. pH is maintained at 3.5-4.5 throughout fermentation. Following incubation, bacterial cells are removed by centrifugation (3,000 rpm, 10 minutes, 3-10 °C), and the supernatant is sterile-filtered (0.22 μm pore size). The final postbiotic formulation contains bioactive metabolites including: antimicrobial peptides (bacteriocins including nisin, plantaricin, lactacin), organic acids (primarily L-lactic acid, D-lactic acid, acetic acid), oligosaccharides (prebiotic compounds), hydrogen peroxide and biosurfactants.

Quality control testing confirms absence of viable bacteria, endotoxin levels < 0.5 EU/mL, and pH 3.8-4.2. Each dose contains standardized concentrations of lactic acid (≥ 50 mM) and bacteriocins (≥ 1,000 AU/mL). Participants self-administered one vaginal applicator (3 mL) daily for 56 consecutive days.

### Vaginal sample collection and DNA extraction

Vaginal samples were collected using sterile flocked swabs (FLOQSwabs; COPAN, Murrieta, CA, USA) from the posterior fornix by trained clinical personnel. Samples were immediately placed in cryovials and transported on ice, then stored at -80°C within 2 hours of collection until batch processing. Genomic DNA extraction and library preparation for PacBio sequencing were performed. DNA samples were quantified using a Qubit 3.0 Fluorometer (Invitrogen, Carlsbad, CA, USA). Amplification of full-length 16S rDNA was carried out using the forward primer 27F with a universal sequence "AGRGTTYGATYMTGGCTCAG" and the reverse primer 1492R with a universal sequence "RGYTACCTTGTTACGACTT." Barcodes were introduced in a second round of amplification, utilizing PacBio Barcoded Universal forward and reverse primers, to generate the libraries suitable for PacBio Sequel sequencing. The DNA input for generating amplicons using the full-length 16S Library Preparation kit ranged from 25pg to 2.5ng.

### Metagenomics sequencing and analysis

AllBio Science, Inc., Ltd. was tasked with conducting the genesequencing and analysis for this study. The sequencing process is briefly as follows, DNA libraries were subjected to validation using the Agilent 2100 Bioanalyzer (Agilent Technologies, Palo Alto, CA, USA) and quantification using the Qubit 3.0 Fluorometer. Following this, the DNA libraries were multiplexed and loaded onto the PacBio Sequel instrument, following the guidelines provided by the manufacturer, Pacific Biosciences of California, Inc., California, USA.

### Gene function prediction

PICRUSt2 was used with the IMG database. Tax4fun2 analysis utilized the official Ref99NR database, and R's microeco package was employed for analysis, plotting, and metastat statistics. FAPROTAX (V1.2.4) analysis was not limited to a specific database and was conducted using R's microeco package for analysis, plotting, and metastat statistics.

### Ovarian stimulation protocol and frozen embryo transfer

All participants received ovarian stimulation using a GnRH antagonist regimen. Baseline assessments consisted of hormonal profiling and transvaginal ultrasonography for antral follicle evaluation. Stimulation was initiated during the early follicular phase (cycle days 1-5) utilizing combined recombinant FSH and LH preparations (Pergoveris, Merck Serono SA, Aubonne, Switzerland). Treatment monitoring involved repeated ultrasound examinations and serial hormone measurements, with gonadotropin doses modified according to individual follicular development.

GnRH antagonist administration (Cetrotide 0.25 mg, Pierre Fabre Medicament Production, Aquitaine Pharm International, Idron, France) began once the dominant follicle measured 12-14 mm and persisted through final maturation. When follicular diameter reached ≥18 mm, final oocyte maturation was triggered using both recombinant hCG (Ovidrel 250 μg, Merck Serono S.p.A., Modugno, Italy) and GnRH agonist (Lupro 2 mg, Nang Kuang Pharmaceutical Co., Ltd., Tainan, Taiwan). Oocyte collection occurred 36 hours post-trigger via ultrasound-guided transvaginal aspiration. Insemination methods (conventional IVF versus intracytoplasmic sperm injection) were determined by sperm parameters and prior fertilization history. All resulting embryos were cultured to cleavage (day 3) or blastocyst stage (day 5/6) and cryopreserved via vitrification using Cryotop method (Kitazato BioPharma, Shizuoka, Japan). This freeze-all strategy was uniformly applied to allow completion of postbiotic intervention before embryo transfer.

Following completion of the eight-week postbiotic intervention, participants underwent hormone replacement therapy cycle for endometrial preparation. On menstrual cycle day 2-3, baseline ultrasound confirmed thin endometrium (< 5 mm) and absence of ovarian cysts. Daily estrogen supplementation included oral estradiol 6-8 mg (Ediol, Synmosa Biopharma Corporation, Hsinchu County, Taiwan) plus transdermal estradiol gel (Oestrogel gel, Besins, Drogenbos, Belgium). Following 14 days of estrogenization, endometrial development was assessed via two-dimensional transvaginal ultrasonography (Voluson E8, GE Healthcare, Chicago, USA). Upon achieving endometrial thickness ≥ 7 mm, progesterone supplementation commenced with three formulations: vaginal progesterone gel 90 mg daily (Crinone 8% gel, Merck Serono, Hertfordshire, UK), oral dydrogesterone 30 mg daily (Duphaston, Abbott, Olst, the Netherlands), and subcutaneous progesterone 25 mg daily (Prolutex, IBSA InstitutBiochimique, Lamone, Switzerland). Embryo warming and transfer timing corresponded to developmental stage: day 4 of progesterone exposure for cleavage-stage embryos and day 6 for blastocysts. Transabdominal ultrasound visualization guided embryo placement during transfer. Progesterone therapy continued through gestational weeks 10-12 in conception cycles.

### Pregnancy outcomes

Clinical pregnancy required visualization of fetal cardiac activity on transvaginal ultrasound at 6-7 weeks' gestation. Ongoing pregnancy denoted continuation beyond 12 gestational weeks. Pregnancy loss was defined as spontaneous termination after cardiac activity detection but prior to 24 weeks' gestation.

### Statistical analysis of species differences

Statistical analyses included Wilcoxon signed-rank test and Welch's t-test using R for inter-group species differences. MetagenomeSeq and ANCOM were used for taxonomic analysis, while ALDEx2 and LEfSe packages were employed for species comparison. Spearman correlation and network analysis were conducted for dominant species. Bubble plots were generated using ggplot2.

## Results

### Patient characteristics and clinical outcome

The baseline demographics and clinical characteristics of the enrolled infertile patient cohort are summarized in Table 1. The cohort was characterized by an advanced maternal age, with a mean age of 38.7 ± 4.1 years. The mean body weight and body mass index were recorded as 62.3 ± 15.3 kg and 23.8 ± 5.5 kg/m^2^, respectively. The mean duration of infertility prior to enrollment was 3.9 ± 2.6 years, with primary infertility constituting the majority of diagnoses at 60% (Table 1). Regarding menstrual parameters, 93% of participants reported a regular cycle, with a mean cycle length of 28.7 ± 2.4 days and a mean menstruation length of 5.7 ± 1.5 days. All fifteen participants completed the full eight-week postbiotic intervention (mean duration 8.0 ± 0.3 weeks) with excellent compliance and no reported adverse events. Following postbiotic treatment and subsequent FET, the cohort achieved a clinical pregnancy rate of 46.7% (7/15) and an ongoing pregnancy rate of 33.3% (5/15). The average time to conception following the initiation of the intervention was 9.1 ± 6.2 weeks.

### Microbial community relative abundance profiles and individual favorable structural shifts

**Figure 1:**
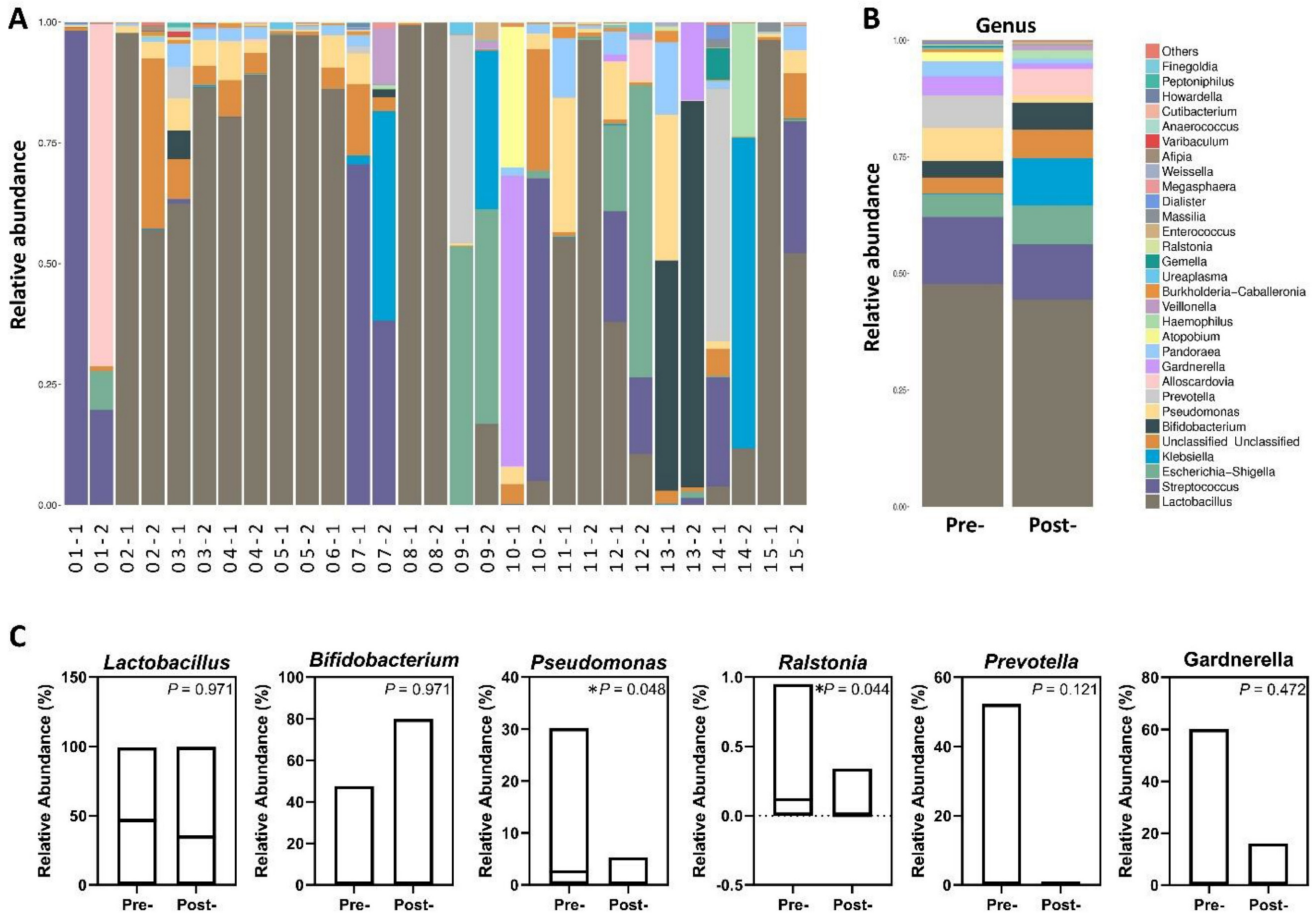
** Vaginal microbial community composition before and after antimicrobial peptide treatment.** (A) Genus-level relative abundance profiles for all individual paired samples. (B) Mean genus-level composition for pre- and post-treatment groups. Taxa are color-coded according to genus classification. (C) Relative abundance comparison of selected genera between pre- and post-treatment samples. Bars represent group means, with corresponding *P*-values from paired statistical testing.

To evaluate patient-specific microbiome responses within this clinically heterogeneous cohort, we generated paired relative abundance profiles at genus level (Figure 1A,1B), with “-1” indicating the pre-treatment sample and “-2” indicating the post-treatment sample collected two months later. Fourteen of fifteen participants contributed paired datasets; sample 06-2 was excluded due to insufficient sequencing quality. Individual stacked bar plots (Figure 1A) revealed pronounced baseline heterogeneity, with participants exhibiting diverse community structures ranging from Lactobacillus-dominant profiles (> 70% relative abundance in patients 04, 05, 08) to complex polymicrobial dysbiotic communities lacking Lactobacillus representation (patients 01, 07, 09, 13, 14). This heterogeneity reflects the variable vaginal microbiome landscapes characteristic of infertile populations with advanced age and recurrent IVF failure. Cohort-mean compositions (Figure 1B) demonstrated that average community structure remained broadly stable, with no dramatic cohort-wide shift following treatment. Quantitative analysis of mean relative abundances (Figure 1C) nonetheless identified significant post-treatment reductions in specific dysbiosis-associated genera: Pseudomonas (P = 0.048) and Ralstonia (P = 0.044) both declined significantly, supporting targeted suppression of detrimental taxa. In contrast, mean Lactobacillus relative abundance showed no significant change (P = 0.977), reflecting the substantial inter-individual variability.

Despite modest group-level changes, systematic examination of paired individual profiles revealed favorable microbiome improvements in 11 of 15 participants (73%), as detailed in Table 2. Two patients demonstrated dramatic Lactobacillus emergence, with patient 03 showing an increase from 62.4% to 86.5% accompanied by near-complete Prevotella clearance (6.5%→0.02%), while patient 11 achieved overwhelming Lactobacillus dominance (55.2%→96.3%) with Pseudomonas near-elimination (27.7%→0.8%). Three patients (04, 05, 08) maintained healthy Lactobacillus-dominant profiles exceeding 88% throughout the intervention with sustained pathogen suppression, demonstrating ecological stability in already-optimized microbiomes. Four patients exhibited pathogen clearance with structural simplification, including patient 09 who increased Lactobacillus from near-zero (0.03%) to 16.8% with complete Prevotella elimination (43.0%→0%), patient 14 who tripled Lactobacillus representation (3.9%→11.7%) with total Prevotella clearance (52.3%→0%), and patients 07 and 13 who achieved ecological purification through near-complete Pseudomonas clearance despite persistently low baseline Lactobacillus levels. Finally, two patients (01, 10) demonstrated modest pathogen reductions with slight Lactobacillus gains, representing partial responses to the intervention. These patient-specific patterns demonstrate that antimicrobial peptide-based postbiotics induced selective microbial modulation tailored to individual baseline dysbiosis profiles, rather than uniform population-wide effects.

### Global microbial community restructuring assessed by β-diversity

**Figure 2:**
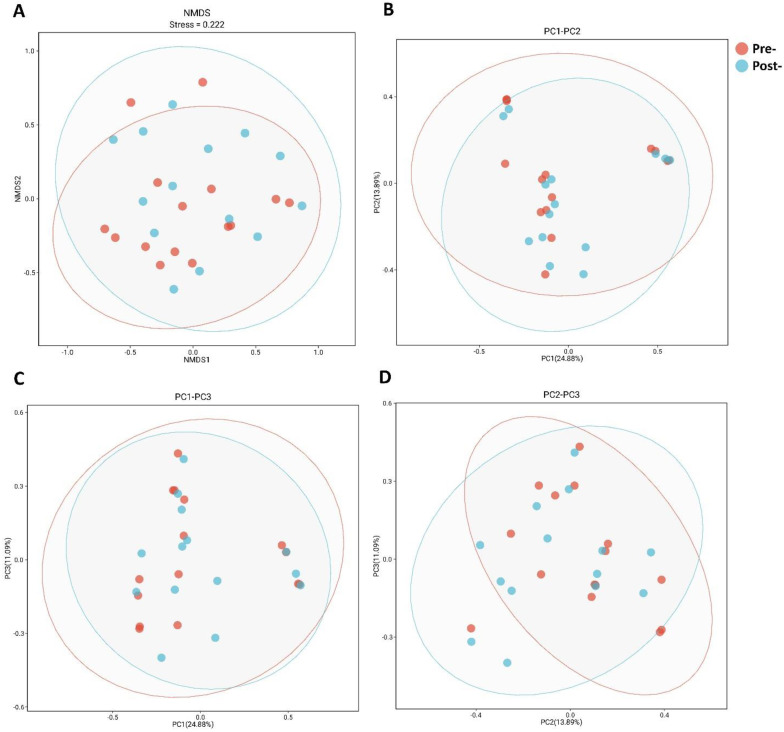
** Ordination of vaginal microbiota β-diversity before and after postbiotics treatment.** (A) NMDS of Bray-Curtis dissimilarities; stress = 0.222; points colored by time point with 95% confidence ellipses. (B) PCoA PC1 versus PC2; percent variance explained by each axis indicated on the plot. (C) PCoA PC1 versus PC3; percent variance explained by each axis indicated on the plot. (D) PCoA PC2 versus PC3; percent variance explained by each axis indicated on the plot.

To assess whether postbiotic intervention induced cohort-wide microbiome restructuring, we evaluated beta-diversity using Bray-Curtis dissimilarities with complementary ordination approaches (Figure 2). NMDS projection (Figure 2A) yielded two-dimensional solution with stress=0.222, within acceptable range for qualitative interpretation. Visual inspection revealed substantial overlap between pre-treatment (red) and post-treatment (blue) samples, with 95% confidence ellipses extensively overlapping and no clear spatial separation. PCoA decomposition (Figures 2B-D) provided variance partitioning across orthogonal axes: PC1 explained 24.88% of variance, PC2 explained 13.89%, with additional variance captured by PC3. Across all three principal component projections (PC1 vs PC2, PC1 vs PC3, PC2 vs PC3), pre- and post-treatment points remained intermingled without consistent directional displacement of group centroids. Formal permutation tests on the full distance matrix did not detect between-timepoint differences in community structure, as indicated by ANOSIM (R = 0.02, p = 0.28) and PERMANOVA (R² = 0.05, p = 0.65). Taken together, these results indicate that eight-week postbiotic intervention did not produce detectable cohort-wide shifts in global community structure, consistent with preservation of microbiome architectural integrity. This finding supports the hypothesis that antimicrobial peptide-based postbiotics exert targeted taxonomic effects rather than broad ecosystem disruption, motivating our focus on taxon-specific abundance changes.

### Identification of differentially abundant taxa with dysbiosis-associated signatures by LEfSe analysis

**Figure 3:**
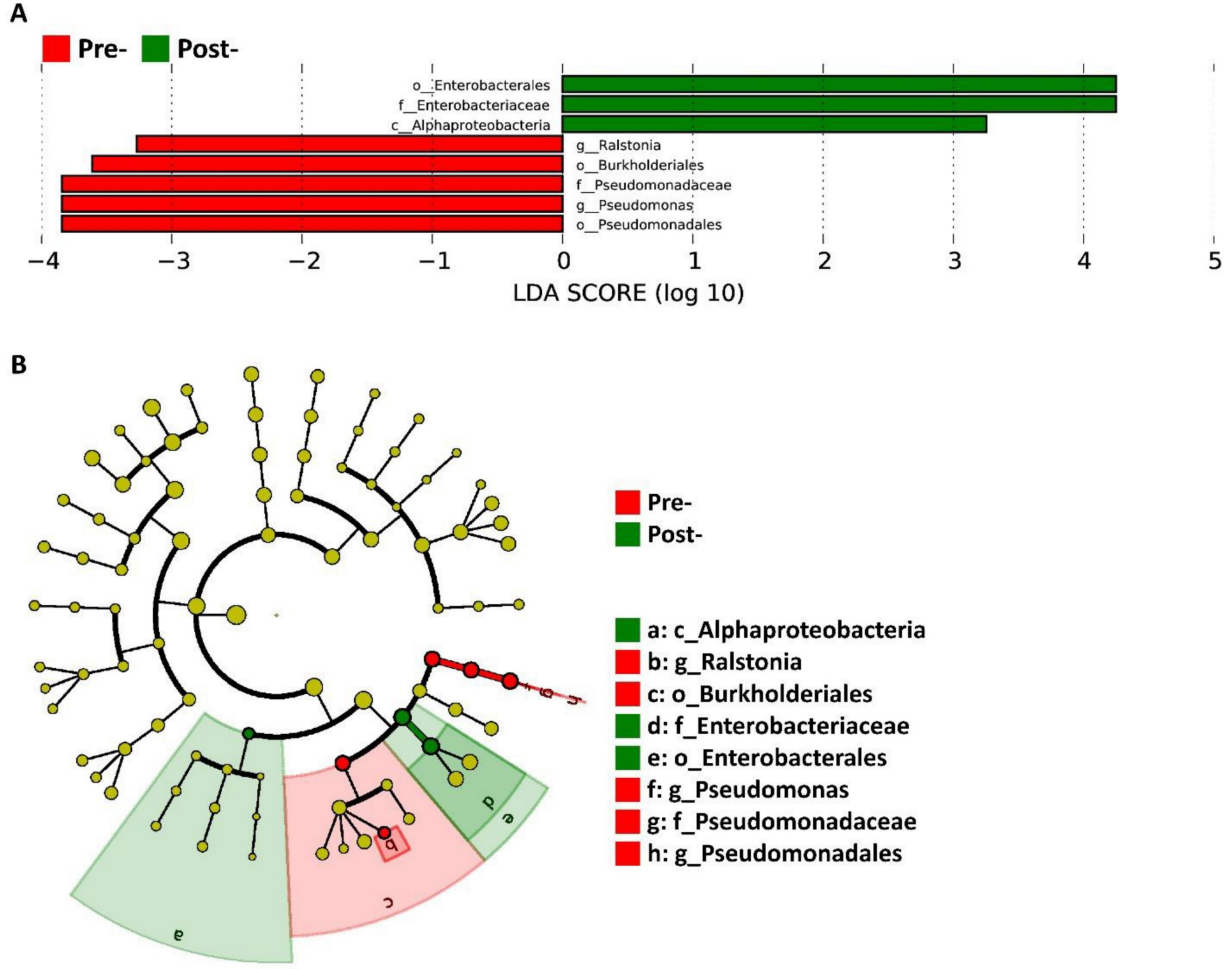
** LEfSe analysis of differentially abundant taxa between Pre and Post.** (A) LDA score plot of taxa enriched in Pre (red) or Post (green); bars indicate effect sizes. (B) Taxonomic cladogram highlighting discriminatory lineages; node colors denote the group in which the taxon is enriched.

To identify microbial features distinguishing pre- and post-treatment microbiota, we performed Linear Discriminant Analysis Effect Size (LEfSe), which integrates statistical significance testing with biological effect size estimation (Figure 3). LDA bar plot (Figure 3A) revealed clear taxonomic separation between timepoints. Pre-treatment samples were enriched for multiple dysbiosis-associated lineages, including *Pseudomonadales*, *Pseudomonadaceae*, *Pseudomonas*, and *Ralstonia*, reflecting the pathogenic and opportunistic signatures characteristic of the baseline microbial environment. In contrast, post-treatment samples showed enrichment of members of *Enterobacterales*, *Enterobacteriaceae*, and the class *Alphaproteobacteria*, indicating a compositional shift in response to antimicrobial peptide treatment.

The taxonomic cladogram (Fig. 3B) provides a phylogenetically organized visualization of these differential features, highlighting the hierarchical positions of taxa contributing to group separation. Branches marked in red correspond to lineages enriched in pre-treatment samples, prominently mapping to the *Pseudomonadales* clade, whereas branches in green correspond to post-treatment-enriched lineages within *Enterobacterales* and *Alphaproteobacteria*. This phylogenetic representation corroborates the LDA findings and illustrates that the discriminatory taxa are localized to distinct, coherent clades rather than dispersed across unrelated lineages. Together, these results demonstrate that the antimicrobial peptide intervention produced targeted taxonomic shifts that align with the patterns subsequently validated through relative abundance measurements.

### Absolute abundance quantification demonstrates targeted clearance of pathogen-associated taxa

**Figure 4:**
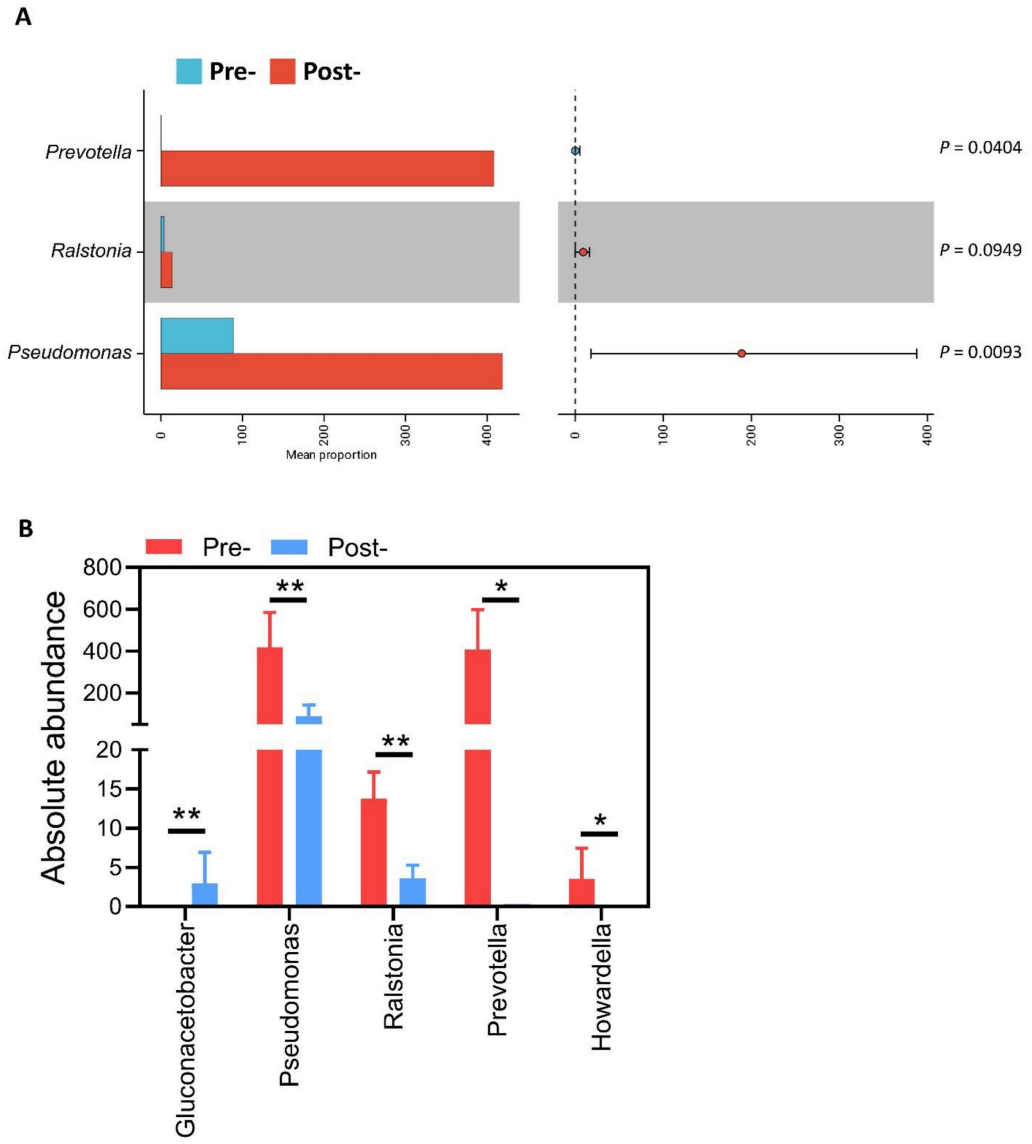
** Absolute abundance of selected genera before and after postbiotics treatment.** (A) Taxonomic distribution of absolute abundance across genera for Pre and Post. (B) Grouped bars showing mean ± SD absolute abundance for *Prevotella, Pseudomonas, Ralstonia, Howardella,* and *Gluconacetobacter* in Pre and Post; q-values indicate FDR-adjusted comparisons of Pre vs Post. *P< 0.05, **P< 0.01.

To validate biological relevance of LEfSe-identified taxa and provide quantitative evidence of antimicrobial effects, we performed absolute abundance measurements for key genera (Figure 4). Taxonomic distribution of absolute abundance across genera (Figure 4A) illustrates overall microbial burden restructuring post-treatment, with notable contraction of high-abundance pathogenic taxa. Quantitative genus-level analysis (Figure 4B) revealed marked selective reductions in dysbiosis-associated organisms. Prevotella demonstrated near-complete elimination from 408.47 ± 990.78 to 0.29 ± 0.83 cells/sample (99.9% reduction, q = 0.0040). Pseudomonas declined substantially from 419.07 ± 567.04 to 89.14 ± 114.42 cells/sample (78.7% reduction, q = 0.0093), while Ralstonia was reduced from 13.80 ± 16.36 to 3.57 ± 7.23 cells/sample (74.1% reduction, q = 0.0949), approaching statistical significance. Most strikingly, Howardella was completely eliminated from 3.53 ± 9.87 to undetectable levels (100% clearance, q = 0.0404). In contrast to the consistent reduction of these detrimental taxa, *Gluconacetobacter* showed a significant post-treatment increase from undetectable levels to 2.93 ± 4.18 cells/sample(q=0.0093). Collectively, absolute abundance data provide quantitative validation that antimicrobial peptide-based postbiotic exerts highly selective effects characterized by substantial reduction of high-load pathogenic genera while preserving overall community stability. This pattern of targeted pathogen clearance without global disruption aligns with favorable individual-level microbiome shifts and supports the precision modulation hypothesis.

## Discussion

This pilot study demonstrates that eight weeks of intravaginal antimicrobial peptide-based postbiotic therapy produces beneficial effects in infertile women with advanced maternal age and recurrent IVF failure: (i) selective clearance of high-burden dysbiosis-associated pathogens including near-complete elimination of Prevotella, substantial reduction of Pseudomonas and Ralstonia, and total Howardella clearance; and (ii) encouraging clinical pregnancy outcomes with 47% pregnancy rate. Importantly, these targeted microbial improvements occurred while preserving global community architecture, as evidenced by stable beta-diversity profiles and absence of wholesale ecosystem disruption.

The selective antimicrobial activity observed in our study likely reflects the mechanistic properties of bacteriocin-enriched postbiotic formulations. VAGINNE^®^ (Good-Care Biotech, Ltd.) contains multiple antimicrobial peptides (nisin, plantaricin, lactacin) with preferential activity against Gram-negative pathogens and anaerobic bacteria characteristic of bacterial vaginosis 24, 25. Unlike broad-spectrum antibiotics that indiscriminately eliminate both beneficial and pathogenic bacteria, bacteriocins demonstrate narrow-spectrum activity determined by specific membrane receptor binding and pore-forming mechanisms 26.

Our finding of near-complete Prevotella elimination (99.9% reduction) carries particular clinical significance. Prevotella species are strongly associated with bacterial vaginosis, produce pro-inflammatory lipopolysaccharides, disrupt epithelial barrier integrity, and correlate with reduced IVF success rates 10, 27, 28. Multiple studies demonstrate that Prevotella-dominated vaginal microbiomes predict implantation failure and early pregnancy loss 29-32. The selective Prevotella clearance achieved without proportional Lactobacillus increase in all patients suggests direct antimicrobial activity rather than simple competitive exclusion. Similarly, Pseudomonas reduction (78.7%) addresses a recognized reproductive pathogen. Pseudomonas aeruginosa produces embryotoxic metabolites, induces inflammatory cascades, and associates with recurrent implantation failure 31. Studies demonstrate that Pseudomonas-positive endometrial or vaginal samples correlate with significantly lower pregnancy rates in assisted reproduction 15, 32. Conversely, the increase in Gluconacetobacter, an acetic acid producer, may represent ecological niche expansion following pathogen clearance, though its functional role in vaginal health requires further study 33.

A key finding is the substantial inter-individual variability in microbiome responses despite standardized intervention. While 73% of participants showed favorable improvements, response patterns varied from dramatic Lactobacillus emergence (patient 11: 55%→96%) to more modest pathogen reduction without Lactobacillus dominance (patients 07, 13). These observations support personalized microbiome-targeted approaches wherein baseline profiling guides intervention selection. Patients with Prevotella- or Pseudomonas-dominated dysbiosis may particularly benefit from antimicrobial peptide postbiotics, while those with extreme Lactobacillus depletion might require combination strategies incorporating live Lactobacillus supplementation.

The 46.7% clinical pregnancy rate observed in our cohort substantially exceeds expected outcomes for women with advanced age (mean 38.7 years) and multiple prior IVF failures. While this single-arm pilot study cannot establish causality, the pregnancy rate improvement magnitude warrants serious consideration and controlled evaluation. Potential mechanisms linking microbiome modulation to improved pregnancy outcomes are multifactorial and interconnected. Pathogen clearance may decrease local inflammatory mediators including TNF-α, IL-6, and IL-1β that impair endometrial receptivity and embryo implantation 34, 35, while simultaneously eliminating dysbiosis-associated proteases and toxins that compromise epithelial barrier function and cervicovaginal immune homeostasis 36. Vaginal pathogen reduction may further decrease endometrial contamination via ascending translocation, particularly significant given the overlap between vaginal and endometrial microbiomes 37, 38. Additionally, Lactobacillus-dominant environments support sperm motility and viability, whereas dysbiotic conditions impair sperm parameters and fertilization potential 39. Collectively, these mechanisms suggest that antimicrobial peptide-based microbiome modulation creates a more favorable reproductive tract milieu through coordinated effects on mucosal immunity, epithelial integrity, ascending infection prevention, and gamete function.

Our findings align with emerging evidence supporting microbiome-targeted interventions in reproductive medicine. Koedooder *et al*. demonstrated that abnormal vaginal microbiota predicts IVF failure, with Lactobacillus-dominant microbiomes conferring 70% pregnancy rates versus 33% in dysbiotic groups 6. Moreno *et al*. showed that non-Lactobacillus-dominant endometrial microbiomes reduce implantation rates from 60.7% to 23.1% 5. However, recent probiotic trials show mixed results. Iwami *et al*. found that vaginal Lactobacillus supplementation improved pregnancy rates in recurrent implantation failure patients 40, while Haahr *et al*. showed no clinical benefit in IVF patients with vaginal dysbiosis 41. Our study extends this literature by demonstrating that postbiotic-mediated pathogen clearance can favorably modulate microbiome composition. Postbiotics may offer advantages over probiotics through delivery of antimicrobial compounds without relying on colonization, enhanced stability and shelf-life, and reduced regulatory concerns regarding live bacterial administration in immune compromised or pregnant populations 22, 23.

Our findings support several important translational applications that warrant further investigation. Microbiome optimization through antimicrobial peptide-based postbiotics represents a modifiable pre-conception intervention with potential to improve outcomes in recurrent IVF failure populations, while baseline microbiome profiling could enable personalized approaches to identify patients most likely to benefit from postbiotic versus probiotic versus antibiotic interventions. Combination strategies pairing postbiotics with live Lactobacillus probiotics may synergistically achieve both pathogen clearance and beneficial bacterial colonization.

Critical future research priorities include adequately powered randomized placebo-controlled trials with live birth as the primary endpoint, correlation of microbiome changes with inflammatory biomarkers and endometrial receptivity markers to elucidate mechanistic pathways, comprehensive metagenomic and metabolomic profiling to identify functional pathways mediating reproductive effects, and long-term safety assessment throughout pregnancy and neonatal development. These investigations will establish whether antimicrobial peptide-based postbiotics merit incorporation into standard assisted reproduction protocols and identify optimal patient selection criteria for targeted intervention.

This study has several important limitations that warrant consideration. The small sample size (n=15) limits statistical power and generalizability, representing a pilot feasibility study requiring validation in larger cohorts. The single-arm design without randomized placebo control prevents definitive causal inference regarding pregnancy outcomes. The relatively short follow-up period captured only clinical pregnancy rates rather than live birth data, which would provide more definitive reproductive outcome assessment. Additionally, the lack of endometrial sampling prevents direct assessment of upper reproductive tract microbiome, though logistical and ethical constraints substantially limit such invasive sampling in clinical practice. Finally, the geographic and ethnic homogeneity of our Taiwanese cohort may limit generalizability to other populations with different baseline microbiome compositions, dietary patterns, and genetic backgrounds, necessitating replication studies across diverse ethnic groups to establish universal applicability.

## Conclusions

This pilot study suggests that eight-week intravaginal antimicrobial peptide-based postbiotic therapy achieves selective clearance of dysbiosis-associated pathogens while preserving global vaginal microbiome architecture in infertile women with advanced maternal age and recurrent IVF failure. The observed 47% clinical pregnancy rate in this population, though requiring validation in randomized controlled trials, suggests promising reproductive benefits warranting further investigation. These findings establish postbiotic interventions as a feasible and potentially efficacious adjuvant strategy for reproductive health, justifying rigorous evaluation in adequately powered, randomized, placebo-controlled trials with live birth as the primary endpoint.

## Figures and Tables

**Table 1:** Patients' characteristics and pregnancy outcomes

Items	Data
Age (years)	38.7 ± 4.1
Body weight (kg)	62.3 ± 15.3
BMI (kg/m^2^)	23.8 ± 5.5
Infertility duration (years)	3.9 ± 2.6
Menstruation cycle	
Regular	93%
Irregular	7%
Menstruation period (days)	28.7 ± 2.4
Menstruation length (days)	5.7 ± 1.5
Type of infertility	
Primary	60%
Secondary	40%
Duration of probiotic supplementation (weeks)	8.0 ± 0.3
Clinical pregnancy rate (%)	46.7% (7/15)
Ongoing pregnancy rate (%)	33.3% (5/15)
Miscarriage rate (%)	28.6% (2/7)
Time to pregnancy following probiotic intervention (weeks)	9.1 ± 6.2

**Table 2:** Patients exhibiting improvement based on relative abundance (%)

Patient Code	*Lactobacillus*(%)		Key Pathogen Change (Psev/ Prev)	Structural Shift Assessment
	Pre-	Post-		
03	62.44	86.45	Prevotella (6.5→0.02) Significant Drop	Significant increase in good bacteria; shift towards Lactobacillus-Dominant CST.
11	55.2	96.3	Pseudomonas (27.7→0.8) Nearly Cleared	Achieved overwhelming Lactobacillus dominance; near-total clearance of major pathogen.
04	80.33	88.93	Pseudomonas (8.0→2.4) Significant Drop	Lactobacillus proportion increased; pathogen relative abundance dropped significantly.
09	0.03	16.83	Prevotella (43.0→0) Total Clearance	Large structural change: Lactobacillus increased from near zero, major pathogen cleared.
14	3.89	11.74	Prevotella (52.3→0) Total Clearance	Favorable shift: Lactobacillus increased, and high initial pathogen load was cleared.
08	99.32	99.83	Pathogens maintained at ultra-low level	Maintained healthy Lactobacillus-dominant structure.
05	97.38	97.31	Pathogens maintained at ultra-low level	Maintained healthy Lactobacillus-dominant structure.
13	0.02	0.02	Pseudomonas (30.2→0.07) Nearly Cleared	Pathogen load significantly cleared (Ecological Purification), despite low Lactobacillus base.
07	0	0	Pseudomonas (6.3→0.02) Nearly Cleared	Pathogens significantly cleared (Ecological Purification).
01	0	0.08	Pseudomonas (0.46→0.02) Drop	Pathogen proportion decreased, minimal structural gain.
10	0.07	5.05	Pseudomonas (3.56→3.18) Slight Drop	Lactobacillus proportion increased, pathogen slightly decreased.

## Data Availability

All data generated or analyzed during this study are included in this published article.

## Abbreviations

CST: community state types
FET: frozen embryo transfer
IVF: *in vitro* fertilization

## References

[1] Mascarenhas MN, Flaxman SR, Boerma T, Vanderpoel S, Stevens GA (2012). National, regional, and global trends in infertility prevalence since 1990: a systematic analysis of 277 health surveys. PLoS Med.

[2] Datta J, Palmer MJ, Tanton C, Gibson LJ, Jones KG, Macdowall W (2016). Prevalence of infertility and help seeking among 15 000 women and men. Hum Reprod.

[3] Gunn DD, Bates GW (2016). Evidence-based approach to unexplained infertility: a systematic review. Fertil Steril.

[4] Ma J, Gao W, Li D (2022). Recurrent implantation failure: A comprehensive summary from etiology to treatment. Front Endocrinol (Lausanne).

[5] Moreno I, Codoñer FM, Vilella F, Valbuena D, Martinez-Blanch JF, Jimenez-Almazán J (2016). Evidence that the endometrial microbiota has an effect on implantation success or failure. Am J Obstet Gynecol.

[6] Koedooder R, Singer M, Schoenmakers S, Savelkoul PHM, Morré SA, de Jonge JD (2019). The vaginal microbiome as a predictor for outcome of *in vitro* fertilization with or without intracytoplasmic sperm injection: a prospective study. Hum Reprod.

[7] Gao X, Louwers YV, Laven JSE, Schoenmakers S (2024). Clinical Relevance of Vaginal and Endometrial Microbiome Investigation in Women with Repeated Implantation Failure and Recurrent Pregnancy Loss. Int J Mol Sci.

[8] Petrova MI, Reid G, Vaneechoutte M, Lebeer S (2017). Lactobacillus iners: Friend or Foe?. Trends Microbiol.

[9] France MT, Ma B, Gajer P, Brown S, Humphrys MS, Holm JB (2020). VALENCIA: a nearest centroid classification method for vaginal microbial communities based on composition. Microbiome.

[10] Onderdonk AB, Delaney ML, Fichorova RN (2016). The Human Microbiome during Bacterial Vaginosis. Clin Microbiol Rev.

[11] Vainamo S, Saqib S, Kalliala I, Kervinen K, Luiro K, Niinimaki M (2023). Longitudinal analysis of vaginal microbiota during IVF fresh embryo transfer and in early pregnancy. Microbiol Spectr.

[12] Su W, Gong C, Zhong H, Yang H, Chen Y, Wu X (2024). Vaginal and endometrial microbiome dysbiosis associated with adverse embryo transfer outcomes. Reprod Biol Endocrinol.

[13] Tian Q, Jin S, Zhang G, Liu Y, Liu J, Tang X (2024). Assessing vaginal microbiome through Vaginal Microecology Evaluation System as a predictor for *in vitro* fertilization outcomes: a retrospective study. Front Endocrinol (Lausanne).

[14] Karaer A, Doğan B, Günal S, Tuncay G, Arda Düz S, Ünver T (2021). The vaginal microbiota composition of women undergoing assisted reproduction: a prospective cohort study. Bjog.

[15] Zhao H, Wang C, Narsing Rao MP, Rafiq M, Luo G, Li S (2025). Effects of vaginal microbiota on *in vitro* fertilization outcomes in women with different infertility causes. Microbiol Spectr.

[16] Moreno I, Garcia-Grau I, Perez-Villaroya D, Gonzalez-Monfort M, Bahçeci M, Barrionuevo MJ (2022). Endometrial microbiota composition is associated with reproductive outcome in infertile patients. Microbiome.

[17] Sehring J, Beltsos A, Jeelani R (2022). Human implantation: The complex interplay between endometrial receptivity, inflammation, and the microbiome. Placenta.

[18] Molina NM, Sola-Leyva A, Saez-Lara MJ, Plaza-Diaz J, Tubić-Pavlović A, Romero B (2020). New Opportunities for Endometrial Health by Modifying Uterine Microbial Composition: Present or Future?. Biomolecules.

[19] Bradshaw CS, Sobel JD (2016). Current Treatment of Bacterial Vaginosis-Limitations and Need for Innovation. J Infect Dis.

[20] Favaron A, Turkgeldi E, Elbadawi M, Gaisford S, Basit AW, Orlu M (2024). Do probiotic interventions improve female unexplained infertility? A critical commentary. Reprod Biomed Online.

[21] Salminen S, Collado MC, Endo A, Hill C, Lebeer S, Quigley EMM (2021). The International Scientific Association of Probiotics and Prebiotics (ISAPP) consensus statement on the definition and scope of postbiotics. Nat Rev Gastroenterol Hepatol.

[22] Vinderola G, Sanders ME, Salminen S (2022). The Concept of Postbiotics. Foods.

[23] Kumar A, Green KM, Rawat M (2024). A Comprehensive Overview of Postbiotics with a Special Focus on Discovery Techniques and Clinical Applications. Foods.

[24] Cotter PD, Ross RP, Hill C (2013). Bacteriocins - a viable alternative to antibiotics?. Nat Rev Microbiol.

[25] Gharsallaoui A, Oulahal N, Joly C, Degraeve P (2016). Nisin as a Food Preservative: Part 1: Physicochemical Properties, Antimicrobial Activity, and Main Uses. Crit Rev Food Sci Nutr.

[26] Bali V, Panesar PS, Bera MB, Kennedy JF (2016). Bacteriocins: Recent Trends and Potential Applications. Crit Rev Food Sci Nutr.

[27] Pelayo P, Hussain FA, Werlang CA, Wu CM, Woolston BM, Xiang CM (2024). Prevotella are major contributors of sialidases in the human vaginal microbiome. Proc Natl Acad Sci U S A.

[28] Muzny CA, Łaniewski P, Schwebke JR, Herbst-Kralovetz MM (2020). Host-vaginal microbiota interactions in the pathogenesis of bacterial vaginosis. Curr Opin Infect Dis.

[29] Liu L, Feng T, Liu Q, Liao M, Liu B, Li M (2024). Characterization of the vaginal microbiota in infertile women with repeated implantation failure. Acta Microbiol Immunol Hung.

[30] Chopra C, Kumar V, Kumar M, Bhushan I (2024). Role of vaginal microbiota in idiopathic infertility: a prospective study. Microbes Infect.

[31] Kumar M, Yan Y, Jiang L, Sze CH, Kodithuwakku SP, Yeung WSB (2025). Microbiome-Maternal Tract Interactions in Women with Recurrent Implantation Failure. Microorganisms.

[32] Chen X, Sui Y, Gu J, Wang L, Sun N (2025). Implication of the Vaginal Microbiome in Female Infertility and Assisted Conception Outcomes. Genomics Proteomics Bioinformatics.

[33] Greenbaum S, Greenbaum G, Moran-Gilad J, Weintraub AY (2019). Ecological dynamics of the vaginal microbiome in relation to health and disease. Am J Obstet Gynecol.

[34] Wei J, Fan X, Zang X, Guo Y, Jiang W, Qi M (2025). LPS Regulates Endometrial Immune Homeostasis and Receptivity Through the TLR4/ERK Pathway in Sheep. Animals (Basel).

[35] Lédée N, Petitbarat M, Chevrier L, Vitoux D, Vezmar K, Rahmati M (2016). The Uterine Immune Profile May Help Women with Repeated Unexplained Embryo Implantation Failure After *In vitro* Fertilization. Am J Reprod Immunol.

[36] Kirthika P, Lloren KKS, Jawalagatti V, Lee JH (2023). Structure, Substrate Specificity and Role of Lon Protease in Bacterial Pathogenesis and Survival. Int J Mol Sci.

[37] Riganelli L, Iebba V, Piccioni M, Illuminati I, Bonfiglio G, Neroni B (2020). Structural Variations of Vaginal and Endometrial Microbiota: Hints on Female Infertility. Front Cell Infect Microbiol.

[38] Polifke A, von Schwedler A, Gulba R, Bensmann R, Dilthey A, Nassar NNR (2024). Differential characteristics of vaginal versus endometrial microbiota in IVF patients. Sci Rep.

[39] Farahani L, Tharakan T, Yap T, Ramsay JW, Jayasena CN, Minhas S (2021). The semen microbiome and its impact on sperm function and male fertility: A systematic review and meta-analysis. Andrology.

[40] Iwami N, Kawamata M, Ozawa N, Yamamoto T, Watanabe E, Mizuuchi M (2023). Therapeutic intervention based on gene sequencing analysis of microbial 16S ribosomal RNA of the intrauterine microbiome improves pregnancy outcomes in IVF patients: a prospective cohort study. J Assist Reprod Genet.

[41] Haahr T, Freiesleben NC, Jensen MB, Elbaek HO, Alsbjerg B, Laursen R (2025). Efficacy of clindamycin and LACTIN-V for *in vitro* fertilization patients with vaginal dysbiosis: a randomised double-blind, placebo-controlled multicentre trial. Nat Commun.

